# Statistical design of Phase II/III clinical trials for testing therapeutic interventions in COVID-19 patients

**DOI:** 10.1186/s12874-020-01101-z

**Published:** 2020-08-31

**Authors:** Shesh N. Rai, Chen Qian, Jianmin Pan, Anand Seth, Deo Kumar Srivastava, Aruni Bhatnagar

**Affiliations:** 1 Biostatistics and Bioinformatics Facility, James Graham Brown Cancer Center, University of Louisville, Louisville, KY 40202 USA; 2 Department of Biostatistics and Bioinformatics, University of Louisville, Louisville, KY 40202 USA; 3 SK Patent Associates, LLC, Dublin, OH 43016 USA; 4 Department of Biostatistics, St. Jude Children’s Research Hospital, Memphis, TN 38105 USA; 5 Department of Medicine, Christina Lee Brown Envirome Institute, University of Louisville, Louisville, KY 40202 USA

**Keywords:** Composite outcomes, COVID-19, Efficacy, Hospitalization, Interim analysis, Intubation, Power, Mortality, Sample size, Toxicity monitoring

## Abstract

**Background:**

Because of unknown features of the COVID-19 and the complexity of the population affected, standard clinical trial designs on treatments may not be optimal in such patients. We propose two independent clinical trials designs based on careful grouping of patient and outcome measures.

**Methods:**

Using the World Health Organization ordinal scale on patient status, we classify treatable patients (Stages 3–7) into two risk groups. Patients in Stages 3, 4 and 5 are categorized as the intermediate-risk group, while patients in Stages 6 and 7 are categorized as the high-risk group. To ensure that an intervention, if deemed efficacious, is promptly made available to vulnerable patients, we propose a group sequential design incorporating four factors stratification, two interim analyses, and a toxicity monitoring rule for the intermediate-risk group. The primary response variable (binary variable) is based on the proportion of patients discharged from hospital by the 15^th^ day. The goal is to detect a significant improvement in this response rate. For the high-risk group, we propose a group sequential design incorporating three factors stratification, and two interim analyses, with no toxicity monitoring. The primary response variable for this design is 30 day mortality, with the goal of detecting a meaningful reduction in mortality rate.

**Results:**

Required sample size and toxicity boundaries are calculated for each scenario. Sample size requirements for designs with interim analyses are marginally greater than ones without. In addition, for both the intermediate-risk group and the high-risk group, the required sample size with two interim analyses is almost identical to analyses with just one interim analysis.

**Conclusions:**

We recommend using a binary outcome with composite endpoints for patients in Stage 3, 4 or 5 with a power of 90% to detect an improvement of 20% in the response rate, and a 30 day mortality rate outcome for those in Stage 6 or 7 with a power of 90% to detect 15% (effect size) reduction in mortality rate. For the intermediate-risk group, two interim analyses for efficacy evaluation along with toxicity monitoring are encouraged. For the high-risk group, two interim analyses without toxicity monitoring is advised.

## Background

### Challenges in COVID-19 clinical trials design

The ongoing COVID-19 (SARS-COV-2 infection) crisis is an unprecedented public health challenge as there are no clinically-proven interventions with substantial evidence that can effectively manage the infection. To meet this challenge, researchers around the world have been working diligently on developing new treatment plans or drugs. Several clinical interventions including those that involve the use of convalescent plasma, a combination of existing drugs, or repurposed drugs, have either entered the clinical trial phase or completed small size studies (a partial list of drugs/therapies used for COVID-19 treatment is given in the appendix). In a recently published preliminary report, *Remdesivir* was deemed to be a better treatment drug in terms of shortening the time to recovery in adults hospitalized with COVID-19, but this drug had previously failed in a relatively smaller trial [1, 2]. In general, COVID-19 research has been criticized for being non-rigorous [3] and many clinical trials have shown uncertain results due to various reasons, including missing or inappropriate control group, small sample size and/or rigorous statistical designs [4–8]. Hydroxyquinone, for example, found to be effective in a small clinical trial [9] failed to show efficacy in a larger trial [10]. Likewise, the trial on Lopinavir-Ritonavir in adults with COVID-19 concluded that “future trials in patients with severe illness may help to confirm or exclude the possibility of a treatment benefit” [8].

Key reasons for the failure of approaches attempted so far are the uniqueness and the range of the population affected by COVID-19 compared with clinical trials with other populations, as well as the speed at which such trials must be conducted. Clearly, there is urgent need for conducting well-designed and well-powered clinical evaluation of potential COVID-19 therapies. However, with patients showing up with a variety of characteristics and fast changing status, it is difficult to recruit and conduct an appropriate trial that could best show the effectiveness of an intervention. Many factors, such as patient status, age, sex, race, co-morbidity, etc., can affect the design or the outcome of the trial and therefore these features must be taken into consideration as stratification factors in designing a well-powered study. Moreover, because of rapid changes in infection rates in a particular location, there is only a limited window of opportunity to conduct single-site clinical trials [11]. Therefore, a wide set of such factors, and a rapidly changing patient population, make it challenging to develop a design that minimizes the imbalance in treatment allocation with respect to stratification factors, while ensuring that the number of strata remain manageable.

### World Health Organization ordinal scale

The World Health Organization has established an ordinal scale system to best describe the clinical status of a patient [12]. A similar 7-category ordinal scale has been used in a previous trial [8]. This scale has proven to be an effective in describing the severity of illness as well as in assessing clinical outcomes in hospitalized patients. This 7-category ordinal scale has been used recently by Wang et al., [13] to categorize outcomes in patients hospitalized with seasonal influenza infection. The authors found the scale to be a useful in capturing a broad range of clinical states as well as in tracking a patient’s status change. Although the ordinal scale is useful for patient classification, but because of differing responses it is not efficient to design a trial based on every stage. Also, patients at different stages of the disease require different treatment. Therefore, we combined groups with potentially similar responses and treatment methods together. The details of this World Health Organization ordinal scale are given in Table 1. Stage 0 is not included here because the uninfected population is not of interest in the context of a clinical trial.

**Table 1:** Different Stages of a Patient

Different Stages of a Patient	
Stage	Condition
8	Death
7	Ventilation with Additional Organ Support (ECMO^a^)
6	Intubation and Mechanical Ventilation
5	Non-invasive Ventilation or High-flow Oxygen
4	Oxygen by Mask or Nasal Prongs
3	Hospitalized; No Oxygen Therapy
2	Limitation of Activities
1	No Limitation of Activities

^a^*ECMO* Extracorporeal membrane oxygenation

### Composite endpoints

A composite endpoint is a single measure of effect, based on a combination of individual components endpoints. Composite endpoints have high utility in evaluating the efficacy of therapeutic interventions that could individually or concurrently alter several different symptoms or outcomes. For example, in Type II diabetics, a drug may affect HbA1C (hemoglobin A1C), body weight, and systolic blood pressure [14]. Often, the frequency of events in individual components of a composite endpoint may be low, so several components are combined to assess the overall efficacy of an intervention. However, each component of a composite endpoint should be clinically meaningful. Ideally, all component should be weighted equally, but this is rarely possible, therefore the relative importance of the components may have to be determined by the frequency of occurrence of the component outcomes. For instance, in cardiovascular trials - death, myocardial infarction (MI), stroke, coronary revascularization and hospitalization for angina are commonly combined, although fatal and non-fatal events are not be treated as the same. In a recent study, patients and clinical trial authors, when asked to assign “spending weights” to five events - death, myocardial infarction, stroke, coronary revascularization and hospitalization for angina, assigned different weights to each of these components [15].

In trials where death is a possible outcome, it is often included as a part of a composite outcome to capture the overall efficacy of the treatment. In this regard, the statistical theory of competing risk supports the inclusion mortality as a component of a composite outcome [16]. In a review of 14 journals between January 2000 to January 2007, of the 1231 cardiovascular trials, 37% used composite endpoints, and 98% of these trials included mortality as a component [17].

In our study design, we selected the 15^th^ day to determine the patient’s status because the 14-day period is the mean duration for patient recovery, or a complete cycle of treatment, as shown in Cao et al. [8]. A 14 day follow up has been used in other studies as well [10]. The estimated mean duration of hospital stay among survivors in the US is 9.3 days (with 95% staying 0.8 to 32.9 days) and among non-survivors was 12.7 days (1.6 to 37.7 days) [18]. Likewise, systemic review of 52 studies, showed that the median length of stay in China was 10–19 days, and 5 (interquartile range: 3–9) outside China [19]. Therefore, a 14-day follow-up is likely to be sufficient for evaluating efficacy. Other useful values (median days) adopted from Cao’s manuscript are:

Time to Clinical Improvement -----------------16 Days

Intensive Care Unit (ICU) Length of Stay------10 Days

Duration of Invasive Mechanical Ventilation (IMV)-5 Days

Days on Oxygen Support------------------------13 Days

Length of Hospitalization------------------------15 Days

The WHO has proposed time to clinical improvement as the primary endpoint in the R&D blueprint report [12]. The “time to clinical improvement” is defined by Cao [8] as the time from randomization to either an improvement of two points on a 7-category ordinal scale or discharge from the hospital, whichever comes first. However, based on conversations with more than 50 frontline physicians, we believe that the WHO endpoint may not be the best choice. The reason is that the authors do not feel that in the intermediate-risk group the time it takes for a patient to be cured, whether it is 15 days or 18 days, is important. What is of greater interest is acceptable recovery from the infection (‘full cure’), therefore, we propose a binary outcome to be the endpoint (whether the patient is cured after 14 days or not). The intent is identify the increase in response rate in the intermediate group with new treatment compared with standard care. The duration to evaluate the efficacy in the high-risk group is short, and there is no censoring, and no loss to follow-up. In the high-risk group, the most important outcome is survival. Therefore, we chose 30 day mortality as the primary outcome and do not suggest using a time-to-event approach for designing the trial.

The endpoints used in other published studies are: time to providing a nasopharyngeal swab negative RT-PCR for SARS-CoV-2 [6], incidence of either laboratory-confirmed COVID-19 or illness compatible with COVID-19 within 14 days [10], reduction in mortality by at least 50% in the high-dose group compared with the low-dose group [5], presence or absence of virus at day 6 [4], all-cause mortality at hospital discharge or at 60 days, and the WHO clinical progression scale [20].

Core Outcome Measures in Effectiveness Trials (COMET) initiative started on May 20-21^st^, 2015 in Calgary, Alberta, Canada. Since then many efforts are underway to develop Core Outcome Set (COS) for various indications including COVID-19 [21, 22]. For COVID-19 disease prevention, efforts are underway to develop COS and for in hospitalized patients with confirmed or suspected COVID-19. Jin et al. have developed COS for mild (time to 2019—nCOV reverse transcription polymerase chain reaction (RT-PCR) negativity), ordinary type (length of hospital stay, severe type (composite events, length of hospital stay, PAO2/FiO2, duration of mechanical ventilation and time to 2019-nCoV negativity), critical type (all-cause mortality) and rehabilitation (pulmonary function) [22]. The WHO group on COS in COVID-19 categorized COS outcome into three categories: Viral burden (PCR or nasopharyngeal swab), survival (All-cause mortality at hospital discharge or at 60 days) and clinical progression - WHO clinical progression scale measured daily over the course of the study [20]. Hospital discharge and mortality are both part of COS for COVID-19. Hospital discharge is around 15 days [23, 24]. The endpoint we have proposed are in line with the COMET initiative.

The purpose of this article is to propose effective statistical designs for COVID-19 clinical trials. Two parallel clinical trials design with respect to different patient risk groups are described. Issues and limitations are discussed. Required sample size in each arm under different scenarios along with toxicity boundaries are calculated and presented in a tabular form for ease of implementation and to inform clinical trial designs.

## Methods

This section contains two subsections, including one design for the intermediate-risk group and the other for the high-risk group.

**Fig. 1:**
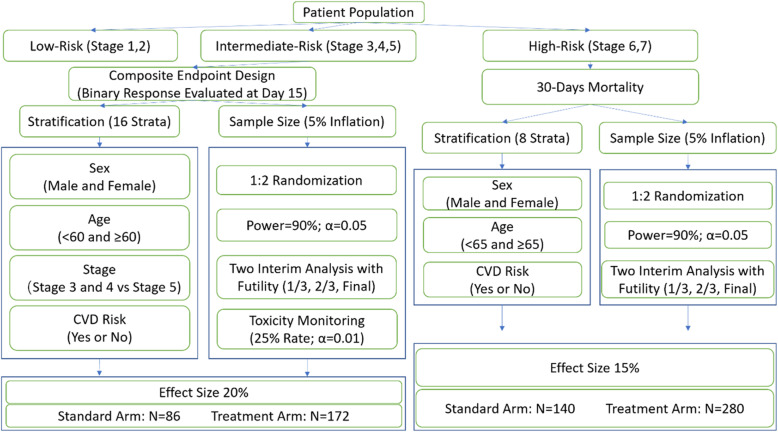
Flowchart of Two Parallel Clinical Trials with Key Design Components

A flowchart that illustrates the overall design of both such trials is shown in Fig. 1.

In our designs, the WHO ordinal scale is used to classify COVID-19 patients into different stages based on their clinical status, but it is difficult to design a trial based on every stage. As a result, we combined groups with similar standard treatment options together. Based on the ordinal scale, patients are separated into three group: High-Risk Group (Stages 6 and 7), Intermediate-Risk Group (Stages 3, 4, and 5), Low-Risk Group (Stages 1 and 2). Patients in intermediate-risk group are treated in a similar way, while the high-risk group patients need more innovative and aggressive treatment. Two independent clinical trials with two different designs are proposed for the intermediate-risk group and the high-risk group. Note that if a patient in the intermediate-risk group could not recover and progresses to the high-risk group, then that patient could be eligible for the high-risk group trial.

We discuss considerations and provide specific justification for five important components when designing both clinical trials, including the outcome of the design, stratification, interim analysis, group ratio, and toxicity monitoring. In addition, futility stopping rules are also considered in both designs since there is no need to spend extra resource and energy if the drug is not effective.

### Design for Intermediate -risk group

#### Outcome variables

Since a larger number of patients are expected in the intermediate-risk group, it is feasible to use binary endpoints (success or failure).

We define *Y* as the primary response variable based on the proportion of patients discharged from hospital by the 15^th^ day. Let *Y = 1* indicate the success outcome if the patient is discharged from the hospital by the 15^th^ day. Let *Y = 0* indicate the failure if the patient is not discharged from the hospital by the 15^th^ day, transiting to a higher WHO scale, or dead. Here, the failure is a composite endpoint. It is the similar logic adopted from the cardiovascular trials mentioned in the background section. In our case, *Y* = 1 is the success with probability *P* and *Y = 0* is the failure with probability 1-*P.* Accordingly, we calculated results based on the improvement of response rate from 40% in the standard arm to various rates (50%, 55%, 60%, 65%, 70%, 75%, 80%) in the treatment arm.

Some secondary outcome variables might also be considered. For example, the change in viral load or biomarkers of inflammation such as ferritin or IL-6, time to reduced viral load, or the number of event-free days in the hospital (event-free survival).

#### Stratification

For ethical reason, group sequential designs are recommended in the current setting. Since many factors could impact the outcome, stratified randomization is more suitable. For doing so, Zelen’s blocked randomization scheme with random block size (randomly selected size 4 or 6) is suggested [25]. In previous work Srivastava et al. [26] found that, with several factors appearing to affect the primary outcome of interest with their true distributions being unknown, or the possibility of causing heterogeneous treatment response among individuals in a group with unknown effect size, stratified randomization approach offered consistently better results if the effect size can be assumed to be marginally similar within each stratum.

Factors, such as age, race, sex, co-morbidity and viral load, which might impact the primary outcomes could be addressed by stratification. However, choosing the right factor for stratification is critically important. Many different issues need to be considered when choosing stratification factors. Based on the current clinical experience showing a strong dependence of COVID-19 outcomes on age, sex and diabetes, obesity and hypertension [27, 28], we consider four such factors for the intermediate-risk group: patient stage, at least one cardiovascular disease risk factor among obesity, hypertension and diabetes (Yes/No), age (< 60 and ≥ 60 years), and sex (Male/Female).

For the intermediate risk group, we further classified patients in the three stages, those in Stages 3 and 4 and those in Stage 5 (essentially classifying patients into those who are not in ICU vs. those who are in ICU) and grouping them into two groups. This is suggested to minimize the number of strata for randomization while ensuring that patients within each stratum are relatively homogenous. All these factors are readily identifiable; however, for defining metabolic syndrome status, it may be necessary to include other factors that are representative of a patient’s health condition. Alternatively, composite risk scores such as the Framingham, Reynolds, or GRACE risk scores may be used. Data to calculate cardiovascular risk score and/or obesity may be readily available, as patients are usually weighed and their blood pressure, cholesterol status, and diabetes are often known upon hospital admission. Although age is usually included in risk factor score, it could also be considered as a separate variable when the risk score cannot be calculated. Assuming that no risk scores are available, in our recommended design, we define two age groups: less than 60 years of age and greater than or equal to 60 years of age. It is generally known that patients in the intermediate-risk group are mostly elderly. Patients less than 50 years of age only count a very small percent of the patients admitted to a hospital. Therefore, the cutoff line at 60 years of age is selected to have balanced strata. Moreover, in view of studies showing that the recovery rate for males is lower than of females [29], sex should also be considered. With these four factors for stratification, there would be a total of 16 strata in the design, which makes the trial design somewhat manageable. Race is not explicitly considered, as there is no indication yet of race-dependent variations in outcome, independent of pre-existing disease burden.

#### Interim analysis

For the intermediate-risk group, two interim analyses are recommended. Results are presented for no interim analysis, one interim analysis and two interim analyses. We describe design parameters at alpha = 0.05 and power = 90%. Because the virus is life threatening, it is important to ascertain the efficacy of the intervention as early as possible and make the drug available to this patient population as soon as possible. Without interim analysis, researchers would know the outcome of the trials only after all patients have been enrolled. If one choose to perform one interim analysis, when 50% of patients are enrolled, then using G-rho spending function with rho equals 2, one would stop the trial at the interim evaluation if the *p*-value of the test for comparing the two groups is less than 0.006 [30, 31]. Additionally, for futility evaluation, trial would also be stopped if the p-value is greater than 0.716. Otherwise, the trial should continue, and the final analysis will be conducted, and the efficacy of the treatment should be declared only if when the p-value is less than 0.047. However, due to the insidious nature of this infection, waiting until 50% of patients enrolled to find out the result may still not be aggressive enough. Therefore, to fast track the process and to ensure that the drug can be made available to those who need it urgently, we recommend two interim analyses, with first interim analysis to be performed when one-third of total patient population has been enrolled and evaluated (effective new treatment if *p* < 0.002) with futility look at *p* > 0.830; second look being performed when two-third of the patients are enrolled and evaluated (effective treatment if *p* < 0.014) with futility look at *p* > 0.298; and the final analysis when all patients are enrolled (*p* < 0.046). Rho equals three is used in the G-rho spending function [30, 31]. The choice of Rho was based on the consideration that we need to make the drug available to the patients quickly but we need to make sure that the trial is stopped early only if we have strong evidence that the drug is effective. This is the reason why we chose the *p*-values cut-offs at interim evaluations to be somewhat conservative (making sure that there is strong evidence in favor of the drug and avoid false positive findings). To explain with an example, assume the overall sample size is 243 in which 81 belongs to the standard care arm and the rest 162 belongs to the treatment arm. At the first interim analysis, we have 54 patients (one third of 162). If *p* < 0.002, then there is strong evidence to declare that the intervention is working, and the trial should stop right away. With this design, researchers can find out early whether the intervention works, or stop, if it is causing unacceptable harm to patients by monitoring toxicities. Considering some unforeseen reasons (e.g., patients change their mind regarding the study after randomization) the sample size should be increased by approximately 5% with resulting total *n* = 256. If the expected effect is somewhat smaller (such as 10%), the sample size will drastically increase (*n* = 978), however, the monitoring rule for efficacy and futility evaluations remains the same.

#### Group ratio

We calculate here sample sizes for both 1:1 and 1:2 randomizations for the intermediate-risk group. However, patients enrolled in treatment arm may be the same or twice the number of patients enrolled in the standard treatment arm. The choice of group ratio depends on the efficacy of the intervention in the pilot studies. If it is a new intervention that has not been approved by the FDA, then 1:1 randomization with block size of 4 is recommended in consideration of patient safety. If it is an approved procedure or drug with some preliminary data on efficacy with known toxicity profile, then 1:2 randomization with block size of 6 is recommended to ensure that if the drug is effective more patients get the advantage of being treated on the more efficacious arm.

#### Toxicity monitoring

Toxicity monitoring is challenging, but necessary. For the intermediate-risk group, a toxicity rate of 25% is recommended due to the urgent need of drugs for treatment. In other words, if intervention provides even minimal improvement, then it should still be considered as there is currently no known drug that is 100% effective against COVID-19. Also note that patient may die during the treatment due to other causes. We recommend monitoring for those toxicities (Common Terminology Criteria for Adverse Events (CTCAE) Grades > 2) that are related, possibly related and probably related to the drug, evaluated by the Data and Safety Monitoring Board (DSMB) [32].

### Design for High -risk group

#### Outcome variables

The number of patients in the high-risk group at each health care facility is likely to be small. Using time-to-survival as an endpoint may not be ideal because the follow-up is short, and it may require a long time to enroll all patients and there would hardly be any right censoring. In other words, very few patients will survive pass the outcome evaluation time, thereby making time-to-survival as an endpoint ineffective. Therefore, in our design we focused on reducing 30 day mortality rate. Note that since there is no censoring and the length of follow-up is very short in this risk group, there is almost no difference between the choices of survival or binary endpoints [33].

We define the 30 days mortality as the primary outcome. Let *Y* = 1 indicate death of patient within 30 days (failure), and *Y* = 0 represents a person still alive on the 30th day (success). Accordingly, we calculated results based on the reduction of mortality rate from 80% or 70% in the standard arm to various rates (70%, 65%, 60%, 55%, 50%, 45%, 40%, 35%) in the treatment arm.

#### Stratification

For the high-risk group, stratification is recommended as sample size is satisfied when using 30 day mortality as primary outcome. Enrolling sufficient patients within a given timeframe should not be an issue assuming the trial is a multi-center trial.

For stratification, similar factors as discussed for the intermediate-risk group design are recommended, with some modification. We consider three factors for the high-risk group: one cardiovascular disease risk factors, such as diabetes, hypertension, and obesity (Yes/No), age (< 65 and ≥ 65 years), and sex (male/female). With these three factors for stratification, there would be a total of 8 strata in the design. The reason of selecting 65 years age as a cutoff point is that in a recent study of COVID-19, mortality rate for those who received mechanical ventilation in the age of 18 to 65 years was 76.4%, and for those over 65 years of age, the mortality rate was 97.2% [34]. Zelen’s blocked randomization scheme with random block size (randomly chose size 4 or 6) is recommended in this risk group [25].

In the high-risk group, we did not further classify patients based on their stages (Stage 6 and 7) as we have done in the intermediate-risk group. The reason is that the number of patients in stage 7 is likely to be small, and it is not possible to stratify based on these two stages. However, technically it is ideal to stratify patients evenly in every arm based on their stages, but that is not achievable in this case. Since we have chosen other factors for stratification, if extreme bias occurs, then stage 7 patients should be dropped, and researchers should only perform analysis on stage 6 patients with 80% power.

#### Interim analysis

For the high-risk group, two interim analyses along with monitoring for efficacy and futility at overall alpha = 0.05 and power = 90% are recommended. The reason is that the mortality rate in these patients is high and they need some innovative treatments. For example, convalescent plasma therapy has been widely attempted among the high-risk group. However, the levels of neutralizing antibodies in specific plasma preparation are likely to vary, leading to variable outcomes. Therefore, if during two interim analyses patients respond better to plasma with specific antibody titers, then the rest of the patients could be moved to the higher quality plasma quickly, so they have a higher chance of survival. In this design, first interim analysis is to be performed when one-third of total patient population has been enrolled, completed 30 days, and evaluated (effective new treatment if *p* < 0.002) with futility look at *p* > 0.830; second look being performed when two-third of the patients are enrolled, completed 30 days, and evaluated (*p* < 0.014) with futility look at *p* > 0.298; and the final analysis when all patients are enrolled and completed 30 days (*p* < 0.046). Rho equals three is used in the G-rho spending function based on the consideration of being more conservative in interim analyses to ensure that the treatment is efficacious and avoid the chances of falsely declaring the treatment to be efficacious, which could mean heavy losses in terms of resources invested and loss of lives. Sample sizes for one interim analysis are also calculated and are given in Tables 8 and 9 in the appendix for reference.

#### Group ratio

For patients in the high-risk group, 1:2 randomization is recommended, because such patients are in danger and possibly have failed other treatments. Hence, they should be treated with whatever intervention available to improve their chances of survival. In addition, sample size is large enough to handle the 1:2 treatment allocation ratio when expecting a reduction of mortality rate from 70% to 55%. Estimated sample sizes for 1:1 randomization are also calculated and provided in the appendix for reference.

#### Toxicity monitoring

For the high-risk group, no toxicity monitoring is necessary since mortality rate (between 70% and 97%) has been reported across many health care facilities. With such a high death rate, it is not necessary to look at the toxicity level. Any reasonable intervention that could increase the chances of saving a patient should be attempted, regardless of treatable toxicities. In addition, two interim analyses are built in our design to help stop the trial early if any harmful events are detected.

## Results

### Result for intermediate-risk group

#### Sample size calculation

In the intermediate-risk group, we assume the baseline success rate is 40% in the standard arm (Standard Care) and increased success rate in the treatment group. This estimate is based on a study showing that 38% patients requiring mechanical ventilation were discharged alive [34]. However, these data are from New York during the most severe phase of the outbreak. The baseline success in this group may be higher and therefore the study design could be modified accordingly. It may also be noted that assuming a baseline response rate of 40% provides a conservative estimate of the samples size and to detect the same effect size (for example, 20%) the power would be enhanced if the baseline response rate is lower than 40%. In other words, sample size would not change much whether the baseline success rate is 30%, 40%, or 50%.

In this section, the design with two built in interim analyses is discussed. Tables with required sample size for no or one interim analysis are provided in the appendix. All values are calculated with one-sided tests and using an un-pooled variance estimate. *EAST* software was used for sample size calculation [31].

Table 2 shows the required sample size for design with two interim analyses. Ideally, improvement of 20% of response rate (justification can be found in the discussion) with 90% power is recommended. For 1:1 randomization, each arm requires 108 patients. In 1:2 randomization, the standard care arm requires 81 patients while the treatment arm requires 162 patients. Comparing this table with Tables 5 and 6 in the appendix, sample size did not increase much from those with no or one interim analysis. Therefore, two interim analyses are recommended since it does not require many additional patients. We suggest inflating the sample size from the table by approximately 5% to account for the loss of information (such as dropout after randomization).

**Table 2:** Required Sample Size for Intermediate-Risk Group Patients with Two Interim Analyses

Required Sample Size for Intermediate-Risk Group Patients with Two Interim Analyses					
Effect size		α = 0.05 with 1:1 Group Ratio		α = 0.05 with 1:2 Group Ratio	
	Power	80%	90%	80%	90%
10%	N1	315	438	235	326
	N2	315	438	470	652
	Total	630	876	705	978
15%	N1	140	194	104	145
	N2	140	194	208	290
	Total	280	388	312	435
20%	N1	78	**108**	58	**81**
	N2	78	**108**	116	**162**
	Total	156	**216**	174	**243**
25%	N1	49	67	37	51
	N2	49	67	74	102
	Total	98	134	111	153
30%	N1	33	45	25	35
	N2	33	45	50	70
	Total	66	90	75	105
35%	N1	23	32	18	25
	N2	23	32	36	50
	Total	46	64	54	75
40%	N1	17	23	13	18
	N2	17	23	26	36
	Total	34	46	39	54

N1: sample size for the standard care arm. N2: sample size for the treatment arm

Response rate = 40%

For 80% power: probability of rejection at each look: 1^st^ look *p* < 0.002, futility look *p* > 0.835, 2^nd^ look *p* < 0.014, futility look *p* > 0.312, final look *p* < 0.046

For 90% power: probability of rejection at each look: 1^st^ look *p* < 0.002, futility look *p* > 0.830, 2^nd^ look *p* < 0.014, futility look *p* > 0.298, final look *p* < 0.046

ρ = 3.0

**Bold** indicates recommended sample size with suggested parameters

#### Toxicity monitoring

Since 1:2 randomization is recommended, we used the sample size value from Table 2 to compute toxicity boundaries (*n* = 81 and *n* = 162) at 25% toxicity level. A summarized toxicity boundary is presented in Table 3. In Table 3, if the overall number of subjects is 5 and out of 5 if there are 2 cases of toxicities, then the trial should stop because the toxicity boundary of 25% is exceeded. *R* computer program was used for toxicity boundary calculation [35]. The full toxicity boundaries can be found in Table 7 in appendix.

**Table 3:** Abbreviated Toxicity Boundaries at Probability of Toxicity = 0.25 and α =0.01

Abbreviated Toxicity Boundaries at Probability of Toxicity = 0.25 and α =0.01	
Maximum Number of Subjects	Number of Subjects with Toxicities
5	2
6	3
8	4
10	5
12	6
14	7
16	8
18	9
20	10
23	11
25	12
28	13
…	…
52	22
55	23
…	…
79	31
82	32
…	…
107	40
110	41
…	…
158	56
161	57
164	58

### Result for high-risk group

#### Sample size calculation

For patients in the high-risk group, sample size is calculated based on different levels of improvement rate at 90% and 80% power with two interim analyses (Table 4). P0 is the 30 days mortality rate in the standard arm and P1 is the 30 days mortality rate in the treatment arm. The sample size is the estimated number of subjects that are required to bring down the mortality rate from P0 to P1. Typically, we recommend a reduction in mortality from 70% to 55% with 90% power. In this case, 133 patients should be enrolled for the standard arm, and 266 should be enrolled for the treatment arm. When conducting two interim analyses, to start with, one should enroll 45 patients for the standard arm and 89 patients for the treatment arm, then perform the first look. Similar procedure should be used for the second and third look. For results with one interim analysis and 1:1 randomization, see Table 8 in the appendix. For results with one interim analysis and 1:2 randomization, see Table 9 in the appendix. For results with two interim analyses and 1:1 randomization, see Table 10 in the appendix. Note that all options have nearly identical required sample size, and therefore, performing two interim analyses would be more cost-effective from risk-benefit perspective such as the increased cost of recruiting additional patients. We suggest inflating the sample size by approximately 5% to account for the loss of information (such as dropout after randomization). As noted in the respective tables, which can be used for other effect sizes, much larger sample size is required if the effect size is smaller.

**Table 4:** Required Sample Size for High-Risk Group Patients with Two Interim Analyses

Required Sample Size for High-Risk Group Patients with Two Interim Analyses												
	Power = 80%						Power = 90%					
	P0 = 80%			P0 = 70%			P0 = 80%			P0 = 70%		
P1	N1	N2	Total	N1	N2	Total	N1	N2	Total	N1	N2	Total
70%	171	342	513	NA	NA	NA	237	474	711	NA	NA	NA
65%	79	158	237	833	1666	2499	109	218	327	1155	2310	3465
60%	45	90	135	213	426	639	63	126	189	295	590	885
55%	30	60	90	96	192	288	41	82	123	**133**	**266**	**399**
50%	21	42	63	54	108	162	29	58	87	75	150	225
45%	15	30	45	35	70	103	21	42	63	48	96	144
40%	12	24	34	24	48	72	16	32	48	33	66	99
35%	NA	NA	NA	17	34	51	NA	NA	NA	24	48	72

P0: 30 days mortality rate in the standard arm. P1: 30 days mortality rate in the treatment arm

N1: sample size for the standard care arm. N2: sample size for the treatment arm

For 80% power: probability of rejection at each look: 1^st^ look *p* < 0.002, futility look *p* > 0.835, 2^nd^ look *p* < 0.014, futility look *p* > 0.312, final look *p* < 0.046

For 90% power: probability of rejection at each look: 1^st^ look *p* < 0.002, futility look *p* > 0.830, 2^nd^ look *p* < 0.014, futility look *p* > 0.298, final look *p* < 0.046

1:2 randomization; ρ = 3.0

**Bold** indicates recommended sample size with suggested parameters

## Discussion

Designing a trial for testing the efficacy of therapeutic interventions for COVID-19 is challenging. The pandemic is new and there is little specific information about the virus and its adverse health effects. We do not yet clearly know the typical course of the infection, the range of susceptibility factors and the effects of co-morbid conditions. How the use of different medications, supplements and pharmaceuticals affect the severity of the infection also remain unknown. Whatever little information we currently have, is constantly being revised as new data become available. Nonetheless, it is important to develop streamlined clinical trial, designed with harmonized measures, questionnaires, biomarkers and clinical endpoints, so that the results of different trials could be compared. This is critically important in current circumstance, where a large number of clinical trials need to conducted, as rapidly as possible and with extraordinary care to ensure that maximal information could be extracted from each trial and the results obtained could be compared meaningfully with other trials in the field to administer effective therapies as soon as possible.

Many factors need careful consideration in designing clinical trials, and critical decisions have to be made regarding which parameters to include and which tests should be conducted. In developing model clinical trial designs here, we gathered information from recently published manuscripts [4–8, 10], while fully recognizing that these may need revision. However, based on currently available evidence, we have developed robust design that may require only minimal modification and updating for rapid implementation.

To aid rapid and robust clinical evaluation, our trials have been designed for feasibility and for minimizing the number of participants required. Even though ideally, for a balanced design many known factors should be considered for stratification, we have selected only the most basic demographic parameters, as too many strata require much larger sample size. On the basis of currently available evidence, the stratification factors considered in our design seem most appropriate and generally-applicable to us; however, investigators should pick the factors that are most suitable for their patients and for the specific requirements of the trial. The stratification factors that we include in our design – age, sex and cardiovascular disease risk seem fundamental to the etiology of the infection, which seems primarily to affect older male individuals with pre-existing cardiovascular disease or cardiovascular disease risk [36].

Reasons for the high susceptibility of individuals with cardiovascular disease risk for COVID-19 remain unclear and are under intense investigation, but it has been speculated that conditions associated with chronic unresolved inflammation – such as diabetes, obesity, cardiovascular disease, which are characterized by intrinsic immune dysfunction leading to inflammation may enhance the risk of severe infection and more severe outcomes [37]. Although there are significant racial and ethnic difference in susceptibility to cardiovascular disease [38, 39], there is little evidence to support racial differences per se and not race-specific differences in cardiovascular disease burden affect COVID-19 severity. However, should emerging data indicate that race is an important determinant of the severity of infection or its outcomes, independent of pre-existing cardiovascular disease risk, it could be used for additional stratification of the patient population. Additionally, if a trial is designed to assess pulmonary or renal outcomes, stratification based on lung or kidney function may be important. Note that it is important to consider stratification factors and balance the randomization so that tests for comparing the two groups would be unbiased.

As an alternative to stratified block randomization, one can use dynamic randomization [40]. In dynamic randomization, more stratification factors can be accommodated. For example, considering site as a stratification factor, assuming many sites are conducting study, the dynamic randomization can be useful. As a hypothetical example with 20 sites, sex, COVID-19 severity (at two level) and a co-morbidity (at two level) as stratification, a dynamic randomization can be used to minimize balance 1:1 allocation within each of 160 strata (20x2x2x2). However, a constant involvement of statistics team and implementation of the allocation approach make the process cumbersome. Also, when the treatment allocation is not equal (1:1), but different (such as 2:1), the probability calculation for allowable difference in allocation becomes much harder. To simplify, when there is frequent communication among sites, it is realistic to drop site as a stratification factor and use a stratified block randomization to implement 1:1 or 2:1 allocation. It is also easier to use a stratified z score to draw the inference.

Table 11 in appendix provides critical values for decision making. The test statistic, Z, is based on the ratio of difference in estimated response rates and corresponding pooled standard error estimate. Because we are proposing a stratified randomized design, the test statistics needs to be estimated within each stratum and then pooled together. It may be noted that testing the equality of two response probabilities can be easily formulated in terms of testing the odds ratio. Then, one can obtain the stratified Z statistic, as described in Srivastava et al., and compare it to the cut-off provided in Table 11 in appendix to decide if the trial should be stopped at an interim analysis [26]. Adjusting for additional covariates or constructing confidence intervals for estimate of effect at interim analyses are not straightforward and requires additional considerations [30].

In the design of our clinical trials, we focused on primary outcomes. In general, mortality as the primary outcome seems appropriate at least for advanced stage patients, while for intermediate risk patients, looking at the proportion of patients discharged from hospital by the 15^th^ day appears more appropriate. However, different primary endpoints may be considered, which along with appropriately selected secondary endpoints could provide important mechanistic information. Current evidence suggests that even though COVID-19 significantly impairs tissue function, much of the tissue injury is mediated by the resultant IL-6-driven cytokine storm that exacerbates pulmonary injury and may further damage other peripheral organs as has been reported for SARS [41–43]. Therefore, an intervention designed to decrease viral load, may be only marginally efficacious in preventing clinical symptoms, even though it might lead to a significant decrease in viral load, after the cytokine storm has already been initiated. Similarly, interventions targeted at pro-inflammatory cytokines (e.g., with antibodies) may not affect the viral load but significantly attenuate the subsequent response and clinical outcomes. Hence, to understand such non-linear relationships between infection and response, it may be important to judiciously select a panel of biomarkers informative of the immune response and its resolution at different stages of clinical disease progression.

In addition to monitoring biochemical, physiological and clinical responses, investigators should also be attentive to toxicity due to the therapeutic intervention per se. However, deciding upon an optimal toxicity monitoring rate is problematic, especially in a patient population with a high death rate, as is the case with advanced stage COVID-19 patients. A systematic toxicity monitoring rule is discussed, and its usage is suggested [44–47]. Usually Dose Limiting Toxicity (DLT) probability is assumed at 33% in Phase I cancer clinical trials, although a limit lower than 33% can be advocated. Yao et al. used a 21% toxicity rate in the previous trial and Ivanova et al. used a toxicity rate of 20% and 25% [48, 49]. Based on our 2-decades of experience in designing clinical trials, we suggest using a toxicity rate of 25%. The assumption here is that toxicities are manageable with some treatment if the patient is cured from COVID-19. Also, many of the drugs that are being evaluated in this population (such as *Remdesvir*) are already approved for another indication by FDA. Codes for calculating toxicity boundaries have been published before [50].

We have designed our clinical trials with the expectation that the treatment or intervention is likely to be more effective than standard care, hence all standard tests in the work were conducted one-sided. This could be readily ascertained during the interim analysis. Nevertheless, in some scenarios where the intervention is clearly not working or causing unacceptable toxicity, it may be appropriate to discontinue the trial and to test a different intervention. But usually this is difficult to establish, and therefore, care should be taken to continue the trial to its entirety, while monitoring closely to higher toxicity rates, particularly in intermediate stage patients.

For the intermediate-risk group, we suggest using a response rate of 20%. If it is desirable to have a lower response rate, for example, 10%, then the trial requires a much larger sample size (around 900 as shown in Table 2), and it is more likely to be a multicenter trial. However, it is usually not cost-effective to have such a large sample size to detect only 10% response rate. Nevertheless, we have provided sample size for different response rate in case someone is interested in conducting trials with response rates other than 20%. Note that when conducting a multicenter trial, no stratification should be conducted on centers.

We suggest inflating sample size very marginally (approximately 5%) from our calculation as normally an inflation of 10% to 20% is performed in regular clinical studies. The reason is that COVID-19 patients are unlikely to be lost in the follow up since could be a lethal disease, and enrolled patients in both the intermediate-risk group and high-risk group are likely to be quarantined in the hospital for an extended period of time. Therefore, we suggest inflation of the sample size by only 5% to account for unexpected events, such as suicide or patient dropout after randomization.

## Conclusions

For the intermediate-risk patient group, we suggest using a composite endpoints design with two interim analyses and four factors stratification. The use of 1:2 randomization is recommended for broader patient benefit. Toxicity monitoring is acceptable at 25% level. For clinical trials with this patient population, we suggest that it is optimal to use 90% power and an improvement of 20% response rate (such as from 40% in the standard arm to 60% in the treatment arm).

For the high-risk patient group, we recommend a clinical trials design targeting the improvement of 30-day mortality with two interim analyses and three factors stratification. Given the precarious condition of patients in this group, no toxicity monitoring is needed. We suggest that for this group, the use of 1:2 randomization is ideal, and that a 15% reduction in the 30 day mortality rate (from 70% in the standard arm to 55% in the treatment arm) may be an optimal measure of acceptable efficacy.

## Data Availability

Not applicable.

## Appendix

### Partial list of drugs/therapies currently under investigation for COVID-19 treatment

Andrographolide (In Silico*,* Molecular Docking).

Antisense RNA (In Vitro).

Bacillus Calmette–Guérin (BCG) (A total 19 Vaccine Clinical Studiess are underway, two in Netherlands, one in Colombia, one in South Africa, two in Egypt, one in India, one in Mexico, one in Australia, one in Brazil, one in Greece, one in Denmark, one in Tunisia, one in France, one in US, one in Canada, 2in Germany and one in Guinea Bissau).

Benzyl-quinazolin-4-yl-amine (Potential Repurpose drug using Bioinformatics).

Bevacizumab (Pilot Clinical trial underway in China).

Camptothecin (Potential Repurpose drug using Bioinformatics).

Carfilzomib (Possible candidate for inhibitory activities against SARS-CoV-2 main protease).

Chloroquine (phosphate) (Clinical trial results published based on a study in China. Eighty-two studies are listed in clinicaltrials.gov as of 07/18/2020).

Convalescent plasma therapy (Clinical study results published. One hundred seven studies are listed in clinicaltrials.gov as of 07/18/2020).

Degarelix (Clinical trial underway in the US).

Didanosine (Potential Repurpose drug using Bioinformatics).

Duvelisib (Phase IItrial underway in the US).

Elbasvir (In Silico, Possible candidate for inhibitory activities against SARS-CoV-2 main protease).

Eravacycline (Possible candidate for inhibitory activities against SARS-CoV-2 main protease).

Favilavir (Approved as an experimental drug in China).

Favipiravir (Clinical trial results published, currently marketed in India and China).

Febuxosstat (FBX) (Clinical trial manuscript accepted).

Human immunoglobulin/Convalescent plasma Therapy (Clinical trial results published, and many are underway)).

Hydroxychloroquine plus Azithromycin (Clinical trial results published; forty-three studies are listed in clinicaltrials.gov as of 07/18/2020).

Interferon lambda (Potential Therapeutic Intervention).

Interferons, Arbidol (Eight studies are listed in clinicaltrials.gov as of 07/18/2020).

Lopinavir/Ritonavir (Clinical trial results published; eighty studies are listed in clinicaltrials.gov as of 07/18/2020).

Macrolides (MAC) (Potential drug candidate).

Methylprednisolone (Clinical study results published. WHO and CDC generally not recommend due to mortality risk and other complications.)

Nitazoxanide (Potential drug candidate with Azithromycin, sixteen studies are listed in clinicaltrials.gov as of 07/18/2020).

R0-90-7501 (Potential Repurpose drug using Bioinformatics).

Remdesivir (GS-5734) (GS-441524) (Clinical trial and compassionate study results published. FDA has approved Remdesvir for COVID-19.)

Sarilumab (Potential drug candidate. Seventeen studies are listed in clinicaltrials.gov as of 07/18/2020).

Tocilizumab (Clinical study results published, fifty-nine studies are listed in clinicaltrials.gov as of 07/18/2020).

Valrubicin (Potential drug candidate).

Vitamin C (Clinical trial underway in Wuhan, China).

Vitamin D (Thirty-one studies are listed in clinicaltrials.gov as of 07/18/2020).

Zinc (Eighteen studies are listed in clinicaltrials.gov as of 07/18/2020).

**Table 5:** Required Sample Size for Intermediate-Risk Group Patients with No Interim Analysis

Required Sample Size for Intermediate-Risk Group Patients with No Interim Analysis					
Effect Size		α = 0.05 with 1:1 Group Ratio		α = 0.05 with 1:2 Group Ratio	
	Power	80%	90%	80%	90%
10%	N1	303	420	226	313
	N2	303	420	452	626
	Total	606	840	678	939
15%	N1	134	186	100	139
	N2	134	186	200	278
	Total	268	372	300	417
20%	N1	75	103	56	78
	N2	75	103	112	156
	Total	150	206	168	234
25%	N1	47	65	35	49
	N2	47	65	70	98
	Total	94	130	105	147
30%	N1	31	43	24	33
	N2	31	43	48	66
	Total	62	86	72	99
35%	N1	22	30	17	24
	N2	22	30	34	48
	Total	44	60	51	72
40%	N1	16	22	13	18
	N2	16	22	26	36
	Total	32	44	39	54

N1: sample size for the standard care arm. N2: sample size for the treatment arm

Response rate = 40%

**Table 6:** Required Sample Size for Intermediate-Risk Group Patients with One Interim Analysis

Required Sample Size for Intermediate-Risk Group Patients with One Interim Analysis					
Effect Size		α = 0.05 with 1:1 Group Ratio		α = 0.05 with 1:2 Group Ratio	
	Power	80%	90%	80%	90%
10%	N1	313	433	234	323
	N2	313	433	468	646
	Total	626	866	702	969
15%	N1	139	192	104	143
	N2	139	192	208	286
	Total	278	384	312	429
20%	N1	77	106	58	80
	N2	77	106	116	160
	Total	154	212	174	240
25%	N1	48	67	37	50
	N2	48	67	74	100
	Total	96	134	111	150
30%	N1	32	45	25	34
	N2	32	45	50	68
	Total	64	90	75	102
35%	N1	23	31	18	25
	N2	23	31	36	50
	Total	46	62	54	75
40%	N1	16	23	13	18
	N2	16	23	26	36
	Total	32	46	39	54

N1: sample size for the standard care arm. N2: sample size for the treatment arm

Response rate = 40%

For 80% power: probability of rejection at each look: 1^st^ look *p* < 0.006, futility look *p* > 0.709, final look *p* < 0.047

For 90% power: probability of rejection at each look: 1^st^ look *p* < 0.006, futility look *p* > 0.716, final look *p* < 0.047

*p* = 2.0

**Table 7:** Full Toxicity Boundaries Table at Probability of Toxicity = 0.25 and α =0.01

Full Toxicity Boundaries Table at Probability of Toxicity = 0.25 and α =0.01	
Maximum Number of Subjects	Number of Subjects with Toxicities
5	2
6	3
8	4
10	5
12	6
14	7
16	8
18	9
20	10
23	11
25	12
28	13
30	14
33	15
36	16
38	17
41	18
44	19
47	20
49	21
52	22
55	23
58	24
61	25
64	26
67	27
70	28
73	29
76	30
79	31
82	32
85	33
88	34
91	35
94	36
97	37
100	38
103	39
107	40
110	41
113	42
116	43
119	44
122	45
126	46
129	47
132	48
135	49
138	50
142	51
145	52
148	53
151	54
155	55
158	56
161	57
164	58

**Table 8:** Required Sample Size for High-Risk Group Patients with One Interim Analysis

Required Sample Size for High-Risk Group Patients with One Interim Analysis				
	Power = 80%		Power = 90%	
P1	P0 = 80%	P0 = 70%	P0 = 80%	P0 = 70%
70%	473	NA	654	NA
65%	220	2235	305	3092
60%	128	575	177	795
55%	84	260	116	360
50%	59	147	81	204
45%	43	94	59	130
40%	32	64	45	89
35%	NA	46	NA	64

P0: 30 days mortality rate in the standard arm. P1: 30 days mortality rate in the treatment arm

N1: sample size for the standard care arm. N2: sample size for the treatment arm

For 80% power: probability of rejection at each look: 1^st^ look *p* < 0.006, futility look *p* > 0.709, final look *p* < 0.047

For 90% power: probability of rejection at each look: 1^st^ look *p* < 0.006, futility look *p* > 0.716, final look *p* < 0.047

1:1 randomization; ρ = 2.0. NA = Not Applicable

**Table 9:** Required Sample Size for High-Risk Group Patients with One Interim Analysis

Required Sample Size for High-Risk Group Patients with One Interim Analysis												
	Power = 80%						Power = 90%					
	P0 = 80%			P0 = 70%			P0 = 80%			P0 = 70%		
P1	N1	N2	Total	N1	N2	Total	N1	N2	Total	N1	N2	Total
70%	170	340	510	NA	NA	NA	235	470	705	NA	NA	NA
65%	78	156	234	827	1654	2481	108	216	324	1144	2288	3432
60%	45	90	135	211	422	633	62	124	186	292	584	876
55%	29	58	87	95	190	285	41	82	123	132	264	396
50%	21	42	63	54	108	162	28	56	84	74	148	222
45%	15	30	45	35	70	105	21	42	63	48	96	144
40%	12	24	36	24	48	72	16	32	48	33	66	99
35%	NA	NA	NA	17	34	51	NA	NA	NA	24	48	72

P0: 30 days mortality rate in the standard arm. P1: 30 days mortality rate in the treatment arm

N1: sample size for the standard care arm. N2: sample size for the treatment arm

For 80% power: probability of rejection at each look: 1^st^ look *p* < 0.006, futility look *p* > 0.709, final look *p* < 0.047

For 90% power: probability of rejection at each look: 1^st^ look *p* < 0.006, futility look *p* > 0.716, final look *p* < 0.047

1:2 randomization; *p* = 2

**Table 10:** Required Sample Size for High-Risk Group Patients with Two Interim Analyses

Required Sample Size for High-Risk Group Patients with Two Interim Analyses				
	Power = 80%		Power = 90%	
P1	P0 = 80%	P0 = 70%	P0 = 80%	P0 = 70%
70%	476	NA	660	NA
65%	222	2250	308	3122
60%	129	579	179	803
55%	84	262	117	363
50%	59	148	82	206
45%	43	95	60	131
40%	33	65	45	90
35%	NA	46	NA	64

P0: 30 days mortality rate in standard arm. P1: 30 days mortality rate in treatment arm

For 80% power: probability of rejection at each look: 1^st^ look *p* < 0.002, futility look *p* > 0.835, 2^nd^ look *p* < 0.015, futility look *p* > 0.312, final look *p* < 0.046

For 90% power: probability of rejection at each look: 1^st^ look *p* < 0.002, futility look *p* > 0.830, 2^nd^ look *p* < 0.015, futility look *p* > 0.297, final look *p* < 0.046

1:1 randomization; ρ = 3.0. NA = Not Applicable

**Table 11:** Stopping Boundaries for Z Statistics and *P*-values

Stopping Boundaries for Z Statistics and P-values										
	Z at 1^st^ Interim	P at 1^st^ Interim	Z at 1^st^ Futility	P at 1^st^ Futility	Z at 2^nd^ Interim	P at 2^nd^ Interim	Z at 2^nd^ Futility	P at 2^nd^ Futility	Final Z	Final P
**Intermediate Risk Group**										
For Design with 1 Interim Analysis	2.539	0.006	−0.571	0.716	NA	NA	NA	NA	1.673	0.047
For Design with 2 Interim Analyses	2.902	0.002	−0.954	0.830	2.199	0.014	0.530	0.298	1.689	0.046
**High Risk Group**										
For Design with 1 Interim Analysis	−2.539	0.006	0.571	0.716	NA	NA	NA	NA	−1.673	0.047
For Design with 2 Interim Analyses	−2.902	0.002	0.954	0.830	−2.199	0.014	−0.530	0.298	−1.689	0.046

All values were calculated based on 90% power

## Abbreviations

COVID-19: Coronavirus Disease
CVD: Cardiovascular Disease
COMET: Core Outcome Measures in Effectiveness Trials
COS: Core Outcome Set
RT-PCR: Reverse Transcription Polymerase Chain Reaction
ECMO: Extracorporeal Membrane Oxygenation
HbA1C: Hemoglobin A1C
MI: Myocardial Infarction
ICU: Intensive Care Unit
IMV: Invasive Mechanical Ventilation
FDA: United States Food and Drug Administration
CTCAE: Common Terminology Criteria for Adverse Events
DSMB: Data and Safety Monitoring Board
DLT: Dose Limiting Toxicity
